# Molecular Pathology of Human Prion Diseases

**DOI:** 10.3390/ijms10030976

**Published:** 2009-03-09

**Authors:** Gabor G. Kovacs, Herbert Budka

**Affiliations:** Institute of Neurology, Medical University of Vienna, and Austrian Reference Center for Human Prion Diseases, Vienna, Austria

**Keywords:** Creutzfeldt-Jakob disease, prion, spongiform encephalopathy

## Abstract

Prion diseases are fatal neurodegenerative conditions in humans and animals. In this review, we summarize the molecular background of phenotypic variability, relation of prion protein (PrP) to other proteins associated with neurodegenerative diseases, and pathogenesis of neuronal vulnerability. PrP exists in different forms that may be present in both diseased and non-diseased brain, however, abundant disease-associated PrP together with tissue pathology characterizes prion diseases and associates with transmissibility. Prion diseases have different etiological background with distinct pathogenesis and phenotype. Mutations of the prion protein gene are associated with genetic forms. The codon 129 polymorphism in combination with the Western blot pattern of PrP after proteinase K digestion serves as a basis for molecular subtyping of sporadic Creutzfeldt-Jakob disease. Tissue damage may result from several parallel, interacting or subsequent pathways that involve cellular systems associated with synapses, protein processing, oxidative stress, autophagy, and apoptosis.

## 1. Definition of disease and objectives of the review

Prion diseases are fatal disorders affecting the nervous system of several species, characterized by: (1) progressive loss of neurons; (2) lack of classical inflammation; (3) appearance of vacuolation in the neuropil (“spongiform encephalopathy”); (4) deposition of abnormal conformers of prion protein (PrP); and (5) transmissibility in most forms of the disease (hence the terminology “transmissible spongiform encephalopathy”, TSE).

In this review, we aim to summarize current concepts in this field: molecular background of phenotypic variability, relation of PrP to other proteins associated with neurodegenerative diseases, and pathogenesis of neuronal vulnerability.

## 2. Phenotypic variability of human prion disease

### 2.1. Variability of the prion protein

There are various forms of PrP; recognition of these is crucial in understanding the phenotypic variability and pathogenesis of prion diseases. The physiological, so called cellular form of PrP (PrP^C^) is detected in the non-diseased brain. This is detergent soluble, sensitive to proteinase-K treatment, and has endogenously truncated fragments. It exists generally in a full length form attached to the cell surface via a glycosylphosphatidylinositol anchor, however, a small amount may be endogenously (C-terminal, N-terminal) truncated, anchorless or cytosolic. It is synthesized in the endoplasmic reticulum and processed in the Golgi apparatus. Its mature form is then carried to the cell surface where most of it is found in lipid rafts [1]. Possible physiological functions of PrP^C^ comprise roles in neurogenesis, synaptogenesis, and neuritogenesis, anti- or pro-apoptotic functions, copper binding, redox homeostasis, and functions of hemopoetic cells [2].

**Figure 1.**
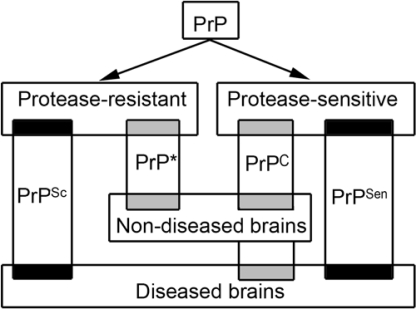
Variability of prion protein (PrP) forms in diseased and non-diseased circumstances. PrP^C^: physiological cellular protease sensitive PrP; PrP*: protease resistant PrP detected in non-diseased brains; PrP^Sc^: protease-resistant disease-associated PrP; PrP^Sen^: disease-associated protease-sensitive PrP.

The major difference between PrP^C^ and the disease–associated form named PrP^Sc^ (where Sc refers to scrapie, a prion disease of the sheep) is a conformational change. PrP^Sc^ features a predominantly beta-pleated structure, while PrP^C^ is alpha-helix dominant [3]. It has generally been thought that PrP^Sc^ is detergent-insoluble and resistant to protease treatment (this form is indicated as PrP^res^ in Western blot studies) [3]. However, a conformation dependent immunoassay also detected protease–sensitive (PrP^sen^) disease-associated transitional forms [4]. The size of disease-specific PrP^Sc^ aggregates ranges from less than 600 kDa to a very large molecular mass [5]. These small PrP^Sc^ aggregates were sensitive to proteolysis [5], moreover, the most infectious units per mass of PrP were demonstrated to be particles of 300–600 kDa [6]. These observations indicate that disease-associated and infectious PrP^Sc^ has protease-resistant and sensitive forms. Indeed, a novel human disease with abnormal PrP sensitive to protease was also recently described [7]. Moreover, PrP^C^ is still present in the diseased brains. The picture is coloured by the recent detection of detergent–insoluble and protease-resistant PrP (designated as PrP*) in non-diseased brains, which may be either non-infectious or may represent dormant infectivity [8,9]. Variability of PrP forms in diseased and non-diseased circumstances is summarized in Figure 1. PrP^Sc^ seems to be the main or only constituent of the infectious agent serving as basis of the ‘*protein-only*’ hypothesis [3,10]. The latter is debated by those arguing for the ‘*not-only-protein*’ hypotheses. Without PrP^C^ there is no prion disease [11].

### 2.2. The spectrum of human prion diseases

Prion diseases may be classified according to etiology, clinicopathological phenotype, and constellation of the PrP gene, *PRNP*, and Western blot characteristics of PrP^res^. Unique to prion diseases is that they may be triggered through infection (inoculation or dietary exposure), germline mutations in *PRNP*, and most frequently by yet unidentified “sporadic” events that generate PrP^Sc^.

Historically, human prion diseases are termed as kuru, Creutzfeldt-Jakob disease (CJD), Gerstmann-Sträussler-Scheinker disease (GSS), and fatal familial or sporadic insomnia (FFI or sFI). Generally, CJD is defined as spongiform encephalopathy, GSS as an encephalo(myelo)pathy with multicentric amyloid plaques, FI as predominantly thalamic degeneration with a relatively characteristic clinical syndrome either lacking or associated with a specific mutation in the *PRNP* (D178N associated with methionin at the polymorphic codon 129) [12,13].

### 2.3. Etiological classification

Acquired forms comprise disorders with suspected or proven external prion exposure. This includes kuru, the disease of the Fore tribe in Papua-New-Guinea related to ritualistic cannibalism; iatrogenic CJD (iCJD), which is related to medical intervention (e.g. neurosurgery, deep electrodes, hypophyseal hormones, dura mater transplants); and variant CJD (vCJD) which represents dietary exposure to bovine spongiform encephalopathy (BSE) [14].

Sporadic forms of prion diseases are sporadic CJD (sCJD) and sporadic fatal insomnia (sFI). In these cases unequivocal demonstration of the source of infection has not yet been achieved (idiopathic).

Genetic or hereditary forms are associated with mutations in the *PRNP* and include genetic CJD (gCJD), GSS, and FFI. Whether GSS has a sporadic (non-hereditary) counterpart still awaits confirmation. Cases with base pair insertions (see below) may present with distinct phenotypes, albeit with features overlapping with other forms. Some authors prefer to designate genetic forms with the mutation and not with historical names. Although many cases present as an autosomal dominant familial disease, lack of positive family history is frequently noted [15,16].

### 2.4. Summary of major clinical presentations

*Sporadic CJD* is generally characterized by progressive dementia, visual or cerebellar disturbance, extrapyramidal or pyramidal dysfunction, myoclonus and in later stages, akinetic mutism. The duration of illness is usually around 3–6 months, and in most cases below 2 years. Most frequently the disease begins at the age of 60–65 years, but teenagers or individuals above 80 may be affected as well. *Iatrogenic CJD* is similar to sCJD, however in cases due to hormone treatment, cerebellar symptomatology may dominate. *Variant CJD* is characterized by early psychiatric symptoms, painful sensory symptoms, ataxia, myoclonus or chorea or dsystonia, and dementia. The median age at onset is 26 (range 12–74) years, and the median duration of illness is 13.0 (6–39) months [17]. Genetic forms of CJD mainly resemble sCJD with longer duration of illness, but atypical presentations may be noted [16]. Clinical symptoms of *GSS* patients may be characterized by prominent, slowly progressive ataxia, and cognitive decline frequently associated with pseudobulbar palsy [18]. In some mutations spastic paraparesis, or parkinsonism may be prominent [16]. In *fatal insomnia* sleep disturbance and autonomic dysregulation are major features, but myoclonus and dementia are also frequent [19].

### 2.5. Neuropathology of human prion diseases

#### 2.5.1. Classical histopathology

**Figure 2.**
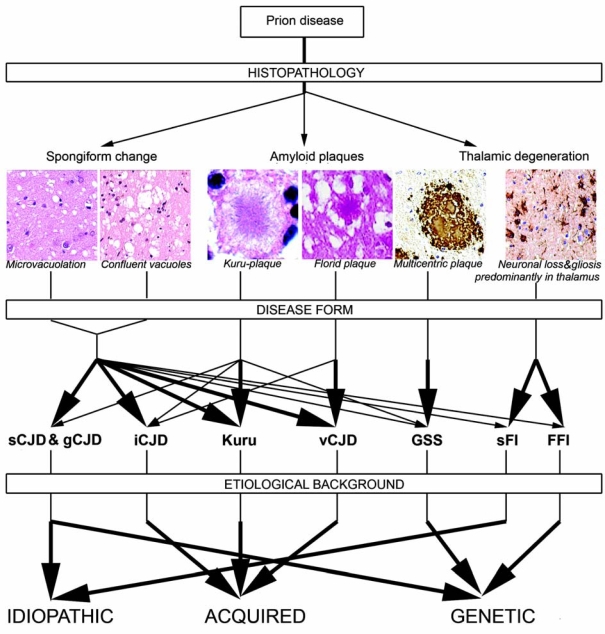
Overview of the spectrum of human prion diseases (microphotograph of florid plaque from a representative case of variant Creutzfeldt-Jakob disease,,courtesy of Professor James Ironside, Edinburgh, UK).

The classical histopathological features of prion diseases include spongiform change, neuronal loss, and astro- and microgliosis. Spongiform change is characterized by diffuse or focally clustered, occasional confluent vacuoles in the neuropil. It should be differentiated from non-specific spongiosis of the brain parenchyma seen in brain edema, metabolic encephalopathies, artefacts, from perineuronal vacuolation in acute/hypoxic damage, and spongiosis of the superficial layers of the cortex in various other neurodegenerative disorders [20]. Amyloid plaques (in particular multicentric plaques) are the prerequisite for the diagnosis of GSS, but morphologically different amyloid plaques are seen in kuru and vCJD. The latter is characterized by abundant amyloid plaques surrounded by vacuoles, designated as “florid plaques” [21]. Amyloid plaques are seen only in a less common molecular subtype (see below) in sCJD. Figure 2 summarizes human prion disease forms, their major neuropathological features and etiology.

#### 2.5.2. PrP immunostaining patterns

Since there is a lack of conformation-specific antibodies suitable for immunohistochemical studies, the detection of disease-associated PrP immunoreactivity requires epitope retrieval methods. In non-diseased brains conventional immunohistochemistry for PrP reveals diffuse neuronal and neuropil immunostaining, which is thought to represent PrP^C^. This should be distinguished from the following immunostaining patterns that are thought to be characteristic for prion diseases and became evident after specific pretreatment of sections [22]: (I) fine deposition (diffuse/synaptic pattern); (II) coarser depositions (these include granules, patchy/perivacuolar - deposits); (III) plaques (with amyloid characteristic, eg. kuru-type and florid plaques, or without amyloid characteristic as plaque-like deposits, focal deposits, or so called microplaques); (IV) vascular amyloid; (V) pericellular deposits as dot-like and/or coarse granular immunoreactivity around unstained neuronal perikarya. In genetic forms of disease additional patterns may be observed [16]. It is important to distinguish PrP immunodeposits that show or that lack amyloid characteristics. The latter, non-amyloid deposits include plaque-like deposits seen only using PrP immunohistochemistry, but not with amyloid stainings (congo-red or thioflavine). Distinction of three major forms of PrP immunodeposits (amyloid versus non-amyloid fine and non-amyloid coarse) correlates well with molecular classification of sCJD cases (see below). In addition this concept helps in understanding the rationale of performing *in vivo* imaging for the detection of amyloid structures. Extremely rarely PrP amyloid may be detected predominantly or exclusively in vessel walls. This was described in a case of stop codon mutation (at codon 145) and also in a single sCJD case [23,24].

And where does disease-associated PrP deposit? Co-localization immunohistochemical and ultrastructural studies [25,26] have indicated that it may be found in synapses, mainly chemical but also electric, in the neuronal cell body and dendrites (thus both post- and presynaptically), furthermore, in intra- and adaxonal localisations. Intracellularly they may be deposited in endosomal-lysosomal structures. Further localization includes macrophages and vascular associated dendritic cells in the vessel wall and perivascular area as well as in astrocytes and microglia.

Deposition of disease-associated PrP is not restricted to the central nervous system in human prion disease. In addition to the involvement of lymphoid tissue in vCJD, other forms may harbour PrP^Sc^ in peripheral nerves, spleen, muscle tissue, neuro- and adenohypophysis [27–31]. Since the presence of PrP^C^ is a prerequisite for the deposition of PrP^Sc^, and the amount of PrP^C^ is low in extraneural tissues, all circumstances leading to an upregulation of PrP^C^, within the frame of its physiological roles, may raise the chance for PrP^C^-PrP^Sc^ conversion. Such a situation may develop in neurogenic lesion of muscle tissue, but more prominently when chronic inflammation or granulomas in different organs lead to increased local production of prions [32–36]. Indeed, this phenomenon was described in a concomitant case of inclusion body myositis and sCJD [37]. Although prion invasion of spleen, lymph nodes and Peyer’s patches depend on lymphotoxin signaling, thus maintenance of follicular dendritic cells, the prion replicating cells within granulomas may be stromal mesenchymal cells, extending the spectrum of cells that may be colonized by prions [32]. In the central nervous system, co-existence of an inflammatory process leads to accelerated death in a mouse model of scrapie [38].

#### 2.5.3. Other proteins in prion diseases

One of the most important aspects during the neuropathological classification of neurodegenerative disorders is the immunohistochemical and biochemical evaluation of protein deposits in the nervous system, which can be deposited intra- and extracellularly [39]. Based on the most important proteins, exemplified by the microtubule-associated tau, alpha-synuclein, amyloid-beta, or TAR-DNA binding protein-43 (TDP-43), diseases are classsified also as tauopathies, synucleinopathies, or TDP-43 proteinopathies. Furthermore, immunostaining for ubiquitin is also of particular interest, since it participates in the degradation of short-lived and damaged proteins. Ubiquitin immunoreactivity is observed in diverse filamentous inclusions of neurodegenerative disorders. The question of the involvement of other proteins than PrP can be raised in two respects; whether other proteins are also present in PrP deposits and how frequently are other proteinopathies associated with prion diseases.

Hyperphosphorylated tau is the major protein in several distinct disorders (tauopathies) and is also an important part of the pathology of Alzheimer’s disease together with amyloid-beta deposition. In prion diseases, phospho-tau deposition has been described in genetic forms (mainly GSS) [18], but is not considered to be part of the neuropathological features of sCJD. Phospho-tau in GSS is thought to be identical to that in Alzheimer’s disease. A recent study reported phospho-tau-immunoreactive neuritic profiles clustered around PrP amyloid deposits in vCJD patients in the absence of amyloid-beta, as well as in mouse models of vCJD [40]. Ultrastructural investigation of amyloid plaques also indicated that florid plaques in vCJD are rather reminiscent of neuritic plaques in Alzheimer’s disease, contrasting kuru plaques and multicentric plaques of CJD and GSS [41]. A recent biochemical study even suggested that the tau protein interacts with PrP [42]. Interestingly, the presumed PrP^C^ immunoreacts with dystrophic neurites and focally co-localizes with early but not fully developed disease-specific inclusions in disorders associated with prominent hyperphosphorylated tau pathology [22,43].

PrP^C^ immunoreactivity is observed not only in the periphery, but also throughout amyloid-beta plaques in Alzheimer’s disease. Whether PrP^C^ is involved the proteolytic processing of Amyloid precursor protein (APP) was adressed recently. Cellular overexpression of PrP^C^ inhibited the beta-secretase cleavage of APP and reduced amyloid-beta formation [44]. Conversely, depletion of PrP^C^ led to an increase in amyloid-beta peptides. These observation indicate that indeed PrP^C^ may be implicated in the development of more frequent neurodegenerative disorders involving the abnormal accumulation of hyperphosporylated tau or amyloid-beta. Indeed, upregulation of PrP^C^ was demonstrated in cortical tissue of various neurodegenerative disorders [43,45].

Granular deposition of alpha-synuclein (a protein marker of Parkinson’s disease, Lewy body dementia, and multiple system atrophy) was also described in sCJD, iCJD and vCJD, mainly in cases with longer duration of illness [46]. These deposits did not regularly co-localize with PrP^Sc^ deposits and with ubiquitin or neural markers. Interestingly, similar findigs were described in an experimental hamster model as well as in scrapie-affected sheep and goats [47].

A further protein identified recently as the major protein in certain forms of frontotemporal lobar degeneration as well as amyotrophic lateral sclerosis, TDP-43, was examined in prion diseases, but failed to support a role of this protein in the pathogenesis of prion diseases [48].

In human prion diseases ubiquitin immunoreactivity was found in a punctate distribution at the periphery of prion protein amyloid plaques and also associated with mainly coarse PrP aggregates [26,49]. In mouse brains infected with the ME7 scrapie strain, ubiquitination of PrP was only detected at the terminal stage suggesting that ubiquitination of PrP is a late event phenomenon and this conjugation occurs after the formation of PrP^Sc^ [50].

In contrast to other neurodegenerative disorders, intracellular ubiquitin immunoreactive inclusions are lacking in prion diseases, although intracellular processing of proteins related to neurodegenerative disorders includes several overlapping components.

The frequency of mixed pathology is rare in CJD compared to other neurodegenerative disorders [51]. A systematic study aiming to assess the co-existence of Alzheimer type-pathology in CJD brains concluded that this most likely represents an age-related change [52].

### 2.6. The concept of strains

Infectious isolates exhibiting distinct incubation times and prion disease phenotype in the same host are defined as prion strains. Strains cannot be encoded by differences in the primary structure of PrP [53]. In conventional pathogens strains are distinguished by differences in their nucleic acid genome. In contrast in prion disease, this is most likely related to different conformational states of PrP that includes also differential proteinase K digestion kinetics [4,9]. Three principal PrP glycoforms are associated with prion strains; both PrP^C^ and PrP^Sc^ exists in three main glycosylation states: mono-, di-and unglycosylated forms [54]. These are widely used as molecular indicators of prion strain typing. To support the notion of strains and also the ‘*protein-only*’ hypothesis, so called synthetic prion strains have been developed and described [10]. The question arises whether these are in fact infectious prions or are simply capable of seeding prion protein production in hosts that have high levels of PrP^C^ and are close to develop a spontaneous disease.

In humans the polymorphism at residue 129 constrains which prion strains may propagate, although the exact mode needs clarification. Diversity of prion strains has been demonstrated in several mammals and has been discussed also in relation with the species barrier [55]. The latter means that prions isolated from one species may be less infectious to other species.

### 2.7. Genetic background of human prion diseases

The human *PRNP*-gene (20p12-ter) encodes a product of 253 amino acids, including octapeptide repeats [56]. Most genetic forms are linked to point mutations (substitution) in the open reading frame. Additionally, a few families carry an insertion in the *PRNP* octapeptide repeat region between codons 51 and 91 [57,58]. In cases with insertional mutation, 2, 4, 5, 6, 7, 8, and 9 plus octapeptides are encoded by 48, 96, 120, 144, 168, 192, and 216 additional base pairs, respectively. The mechanism of insertional mutation may be described as a multi-stage replication strand slippage. In contrast to trinucleotide repeat diseases, inserted mutations remain stable through generations. Although traditionally cases are classified either as CJD or GSS phenotype, it must be noted that both clinically and neuropathologically cases with insertional mutations may be atypical [16,59].

In addition to mutations, polymorphisms with or without established influence on phenotype are known. The role of the polymorphism E219K was suggested upon population based studies in Japan, since the ratio of heterozygotes is higher in the non-diseased [60]. Codon 129 is the best investigated polymorphism. Here either methionine or valine is encoded, thus, depending on the variability of alleles, individuals may be either methinone/valine (MV) heterozygotes, or MM, VV homozygotes. This polymorphism influences the phenotype and susceptibility in human prion disease forms [61–65]. Susceptibility is related to homozygosity and may be exemplified by the fact that 100% of vCJD cases with analysis of the *PRNP* are MM homozygotes. In the normal population heterozygotes represent 50%, MM 39% and VV 11%. In contrast, in sCJD cases MM homozygotes represent 70% (64–81% in different studies). It is striking that 41% of patients younger than 49 years are VV, while 84% of patients above 80 years are MM homozygotes [61]. Noteworthy is the observation that population data from non-diseased Japanese people revealed a much higher prevalence of MM homozigosity (164/179) [66]. In genetic forms the codon 129 polymorphism may influence duration of illness and age at onset (e.g. D178N, 144 base pair insertion) [67–69]. A further eminent instance concerns FFI and gCJD, both with the mutation D178N; when the mutated allele of D178N encodes methionine at codon 129, FFI will be observed, with highly selective vulnerability of the thalamus and lack of spongiform change, in contrast when valine at codon 129 is encoded with prominent neocortical spongiform change [67].

The codon 129 polymorphism was investigated in several disorders; in schizophrenia, mesial temporal lobe epilepsy, and frontotemporal lobar degeneration; an unequivocal influence was not demonstrated, while some studies indicate that in physiological activities like sleep, in ageing brain or in Alzheimer’s disease, the presence of a valine allele may have some role, although this is debated by others [70–79].

Downstream of *PRNP* is the *Doppel (PRND)* gene encoding a 179 aa protein [80]. In a certain prion protein gene knockout animal models an ectopic and increased expression of *PRND* is observed associated with loss of Purkinje cells and ataxia that can be rescued by deletion of *Doppel* from PrP knockout mice [81]. Further downstream is the testis specific *PRNT* gene. The function and relation to prion disease of the latter, together with the *SPRN* gene (10q26.3; shadoo: ”shadow of prion protein”), needs further clarification [80,82]. Some studies aim to clarify whether other genes have influence on prion diseases. One gene is apolipoprotein E; some studies indicated that the ɛ4 allele carries a risk, while others indicated that the ɛ2 allele is a prognostic factor [83–85]. Polymorphisms in the doppel or *ADAM10* gene were not clearly influential either [86,87]. Others reported that polymorphisms in the upstream region of *PRNP* exon 1 may carry a risk for sCJD [88]. Recently, an increased proportion of TT homozygotes for the cathepsin D C224T polymorphism was reported in vCJD patients [89]. The influence of genes like Apolipoprotein E or cathepsin D may modify concomitant pathology (e.g. Alzheimer-type) and thus alter the clinical presentation.

### 2.8. Molecular classification of human prion diseases

It is well known that sCJD may present with a variety of clinical and histopathological phenotypes suggestive of the presence of prion strains in human disease. In addition, molecular prion strain typing was one indicator that vCJD is a distinct form of human prion disease [90]. Early observations indicated that the codon 129 polymorphism is one influential factor. This was exemplified also by differences of PrP immunoreactivity patterns in the cerebellum in sCJD [91]. A landmark study of 300 individuals affected by sCJD by Piero Parchi and Pierluigi Gambetti confirmed that the codon 129 polymorphism in combination with the Western blot pattern of PrP^res^ serves as a basis for molecular subtyping of sCJD [54]. Based on differences in electrophoretic mobility and N-terminal sequence of the core fragments, originally two forms of PrP^res^ were distinguished. Type 1 has a relative molecular mass of 21 kDa, and type 2 of 19 kDa. Thus according to codon 129 constellation and PrP^res^ type, sCJD is classified in at least six groups as MM, MV, VV with PrP type 1 or type 2, respectively [54]. Soon it was demonstrated that vCJD shows type 2 PrP^res^ but with a different glycoform ratio designated as type 2B, to be distinguished from type 2A in other forms (hence vCJD is MM type 2B) [22,92]. Another group identified four major types of PrP^res^ on Western blots from human prion diseases. Type 1 according to Parchi and Gambetti corresponds to type 1 and 2 by Collinge, while type 2A is type 3, and type 2B is type 4 [90,93]. The background of differences was revisited recently. It was proposed that the variability of the molecular mass of PrP^res^ underlying the division of sCJD MM type 1 into two subtypes is most likely due to pH variations during tissue preparation. Those authors suggested that differentiation of sCJD MM type 1 into two subgroups is not justified [94].

A further issue relates to the co-occurence of PrP^res^ types 1 and 2 in the same brain. This phenomenon was already reported in the first large study and was subsequently confirmed by others [54,95,96]. Using antibodies presumed to recognize type 1 PrP but not type 2, patients classified as sCJD type 2, as well as vCJD cases, showed variable amounts of PrP^res^ that matched type 1 in gel migration characteristic [97,98]. This could mean that the two major PrP types exist in a dynamic equilibrium within the brain and would challenge the molecular typing of CJD based on Western blot signature patterns. However, others argued that this phenomenon may not be the rule and may have a methodological background. Using high-resolution electrophoresis and a wide range of proteinase-K treatment indicated that there are many type 2 CJD cases lacking evidence for co-occurrence of type 1 PrP^res^ [99]. In conclusion, it seems plausible that 1) the original 6 subtypes of sCJD proposed by Parchi and Gambetti represents a reliable molecular classification of cases; 2) some but not all brains (around 15–20% of cases) may contain a mixture of type 1 and type 2 PrP^res^ that may influence the phenotype; 3) vCJD harbours a well distinguishable type of PrP^res^.

**Figure 3.**
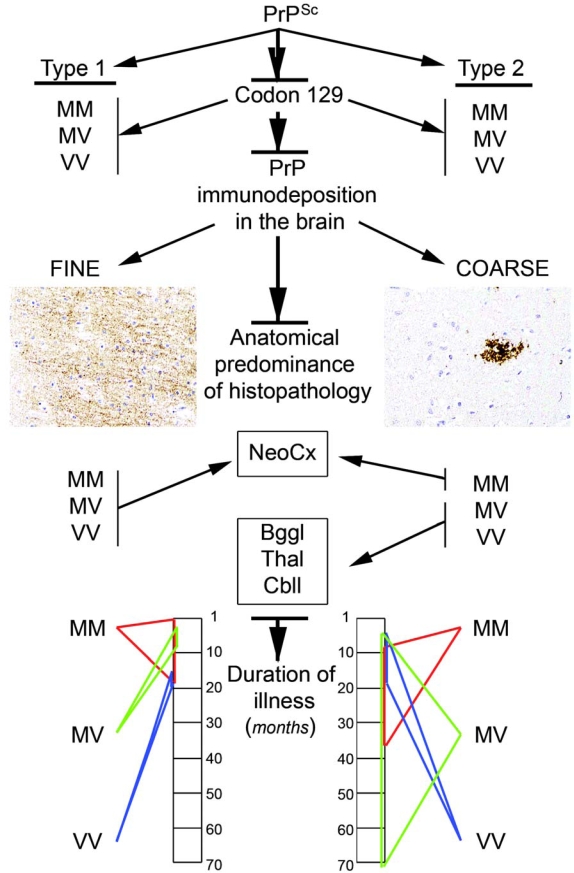
Differences between molecular subtypes of sporadic Creutzfeldt-Jakob disease. M: methionine; V: valine; NeoCx: neocortex; Bggl: basal ganglia, Thal: thalamus, Cbll: cerebellum.

In sCJD patients presence of type 2 PrP^res^ is generally associated with a longer disease course, different anatomical involvement of the brain, and particularly difference in the immunomorphology of PrP deposits. Type 2 PrP^res^ is represented by more aggregated coarser deposits including plaques in contrast to fine appearance of PrP immunoreactivity associated with type 1 PrP^res^. Further phenotype-modifying effects of the codon 129 polymorphism are evident (Figure 3).

These PrP Western blot patterns are detectable also in genetic forms of human prion disease. Some studies have shown that the glycoform ratios associated with *PRNP* point mutations are distinct from those observed in sporadic, iatrogenic and variant CJD [100]. Patients with the same *PRNP* mutation can also propagate PrP^Sc^ with distinct conformations providing insight into the diverse clinicopathological phenotypes. GSS has an additional biochemical marker represented by low molecular weight bands further supporting the notion that GSS is a distinct form of prion diseases (“PrP amyloidosis“) [101,102].

Recently a novel human disease was described, which shows a distinct clinical and neuropathological phenotype. Since typical PrP^res^ was not detected, these cases were designated as protease-sensitive prionopathy (PsPr) [7].

## 3. Neuronal degeneration in prion disease

The cellular pathogenesis of human prion diseased was extensively reviewed recently [103]; here we summarize the major processes that are important to understand the neuropathology of prion diseases.

### 3.1. Pathways of prion transport

The routes of infection in natural and experimental prion diseases comprise uptake of prions via the alimentary tract or through scarification of gums, skin, and conjunctiva, or intracerebral, intraperitoneal, intramuscular, or intravenous inoculation. Spread of the agent depends on their site of entry, strain, dose, and species and PrP genotype of the host [104].

The following cellular and subcellular components are implicated during these processes: 1) After peroral challenge, the agent accumulates in lymphoid tissue, in particular gut-associated lymphoid tissue and draining lymph nodes, spreads to the peripheral nervous system, travels up to, and disseminates within, the central nervous system, and then finally spreads to peripheral sites [104]. B cells and follicular dendritic cells are thought to have an important role in this process [105,106]. 2) For prion propagation in the nervous system, axonal transport, passive translocation in perineural lymphatics, spread in neural interspaces, and a domino-like conversion of PrP^C^ into PrP^Sc^ along neural cell membranes have been proposed [26,104,107]. 3) Further modes of transport involve macrophages and dendritic cells.

### 3.2. Cell death pathways in prion disease

Synaptic degeneration and loss are suggested to precede neuronal degeneration, in particular since both PrP^C^ and PrP^Sc^ locate to synapses. Early and more recent sequential studies revealed progressive loss of dendritic spines [108]; possibly Notch-1 may be a mediator of this process [109]. Loss of synapses and dendritic spines from an early stage in the disease process may have the effect of isolating neurons from electrical stimuli and trophic factors [108,110].

In various forms of prion diseases, demonstration of morphological features of apoptosis, DNA fragmentation, and activation of caspase-3 supports apoptosis as a relevant cell death pathway in prion disease. The variability of results suggests that this is not exclusive. Another cell death process, autophagy, is also present as demonstrated by autophagic vacuoles in experimentally induced scrapie, CJD, GSS, and FFI [111]. Further studies showed that oxidative stress is a global event in prion disease affecting various types of neurons, while there seems to be some selective neuronal vulnerability (e.g. of parvalbumin immunoreactive GABAergic neurons) [112].

Based on the central event of protein conversion in prion diseases, several studies on pathogenesis investigate protein-processing systems, like the ubiquitin-proteosome system (UPS), chaperones, and the endosomal-lysosomal system (ELS). Neuronal cells overexpressing PrP^C^ develop cytosolic PrP^C^ aggregates under conditions of mild proteosome inhibition that did not cause cell death. Neuronal propagation of prions invoke a neurotoxic mechanism with intracellular formation of compartmentalized cytosolic PrP^Sc^ aggresomes [113]. Moreover, disease associated PrP oligomers inhibit the proteasome [114]. In human diseased brains cytosolic PrP^Sc^ aggregates have not yet been observed, distinguishing prion diseases from several other neurodegenerative disorders, where abnormal proteins aggregates are found intracellularly. However, the nuclear redistribution and accumulation of UPS components in sCJD, in correlation with regional tissue damage and demonstration of ubiquitin immunoreactivity in extracellular PrP deposits, support the notion of the involvement of the ubiquitin-proteosome system [115]. Distribution of neuronal immunostaining for ubiquitin, proteasomal subunits, and the inducible heat shock protein Hsp72 in non-diseased control brains suggests that “weakest-links” do exist in the brain and may be predisposed to neuronal damage after a yet unidentified initiating event. Chaperones, including heat shock proteins may promote or inhibit the formation of the pathogenic conformation. In addition, they might build up a cellular defense response and may contribute to save PrP^C^ [116]. In fact, one important component of protein folding homeostasis is the heat shock response that has implications for prion disease. Heat shock factor 1 knockout mice infected with RML prions show similar kinetics of PrP^res^ accumulation, but they have a dramatically shortened lifespan, supporting the protective role of heat shock response [117]. The endosomal-lysosomal system is involved in the processing of both PrP^C^ and PrP^Sc^, and it may even be the site of PrP^C^–PrP^Sc^ transformation [2,25,118,119]. PrP^Sc^ may also end up in mature lysosomes for degradation by proteases. Indeed we demonstrated that accumulation of the lysosomal enzyme cathepsin D correlates with regional pathology [120]. Overloading of this system with undegradable PrP^Sc^ might result in the failure of lysosomal functions and cytopathological consequences.

Involvement of different forms of PrP in the pathogenesis has several aspects. Most likely a loss-of-function of PrP^C^ (e.g. antioxidant and antiapoptotic functions) may be exacerbated by additional toxic gain-of-function of PrP^Sc^ and influenced also by further forms of PrP (e.g oligomeric) [1]. A recent study suggested complex interaction of the presumed toxic activity of PrP^Sc^ and the protective activity of PrP^C^. It was theorized that the interaction of PrP^C^ with its putative coreceptor is modulated by the formation of a PrP^C^/PrP^Sc^ complex, leading to a switch from stress-protective to pro-apoptotic signaling [121]. The issue whether toxic gain-of-function or loss-of-function mechanisms are responsible for pathogenicity has major therapeutic implications. In this respect, some features of illness associated with PrP mutations were suggested to derive from a loss of neuroprotective function of PrP, in contrast with a toxic gain-of-function mechanism [122]. The GPI anchor is an important component of PrP. Lack of this GPI anchor is associated with the presence of more PrP^Sc^ in an amyloid form, and this leads to less infectivity and neurodegeneration [123]. Transient non-PrP^Sc^ neurotoxic components may be also considered for the pathogenesis [124]. Further forms of PrP have also implication for the pathogenesis. Toxicity of PrP located in the cytosol was discussed and reviewed recently [125,126]. These toxic PrPs most likely exert their damage through interactions with a variety of proteins. An example is the observation of an association of cytosolic PrP with the anti-apoptotic protein Bcl-2, a process modulated by cytosolic chaperones including Hsp70 [127].

The complex cellular pathogenesis of prion diseases is supported by further neuronal as well as glial pathways. Terminal complement activation was demonstrated in human prion disease [128]. Complement activation may lead either to sublytic levels of C5b-9 that may generate oxidative stress and induce apoptotic cell death, or to direct cell lysis with consecutive tissue damage. The role of the membrane attack complex as a decisive factor for neuronal death was questioned in an experiment with C5-deficient mice [129]. Endoplasmic reticulum response to cellular stress conditions, termed unfolded protein response, is considered in the pathogenesis of neurodegenerative disorders. In human brains PERK, which launches the most immediate response to ER stress, were not found to be activated in human and experimental prion diseases, in contrast to Alzheimer’s disease [130]. Indeed, in an XBP-1 (X-box-binding protein, a key transcriptional regulator of the unfolded protein response) knockout mice infected with murine prions neither prion aggregation, neuronal loss, nor survival was affected [131].

Astrocytosis and microglia activation is a prominent feature of prion diseases [103]. Upregulation of astrocytic enzymes follows the rise in PrP, suggesting that the astrocytic response is induced by PrP^Sc^. Studies suggest that microglia may process or degrade PrP^Sc^. An interesting aspect of astroglia and microglia response was highlighted in the chemokine receptor CXCR3 deficient mice infected with prions [132]. In this model microglia activation was attenuated together with excessive astrogliosis and accelerated accumulation of PrP^Sc^, whereas survival times were significantly prolonged; thus a novel disease-modifying host factor related to micro- and astroglia response was identified.

As briefly summarized above, many proteins and pathways have been implicated in the pathogenesis of prion diseases. Analyses of gene expression have been performed in order to show what might be important. Inactivation, absence, or overexpression of a gene may affect prion replication if it contributes to pathogenesis. A recent systematic study tested 20 candidate genes and suggested that most do not significantly affect survival times, whereas ablation of amyloid precursor protein or interleukin-1, and overexpression of human superoxide dismutase 1, prolong incubation times between 13–19% [133]. These observations also emphasize a general fact in research on prion diseases, that the genetic background of the animal model, the prion strain, inoculum titers, and route of infection may all impact on the results that thus must be interpreted with caution when considering for relevance to human prion diseases.

## 4. Summary

The following features characterize human prion diseases:

1. PrP exists in different and overlapping forms in diseased and non-diseased brains; however, abundant disease-associated PrP together with tissue pathology characterizes prion diseases and associates with transmissibility.
2. Different etiological forms of prion disease differ in pathogenesis, which has implications for public health, clinical differential diagnosis, research, spread of infectivity within the body, and also therapeutic approaches.
3. In diseased brain PrP is the major protein that deposits mainly extracellularly in the brain; however, other proteins associated with other neurodegenerative disorders, in particular hyperphosphorylated tau, amyloid-beta, and alpha-synuclein may be deposited as well. The exact interactions of these proteins await clarification.
4. Mutations of the *PRNP* are associated with genetic forms; however the polymorphism at codon 129 has a crucial influence on phenotype and susceptibility and may have implications for other non-prion disorders.
5. The codon 129 polymorphism in combination with the Western blot pattern of PrP after proteinase K digestion remains as a basis for molecular subtyping of sCJD. This represents strains in human prion disease.
6. Tissue damage may result from several parallel, interacting or subsequent pathways that involve cellular systems associated with synapses, protein processing, oxidative stress, autophagy, and apoptosis.
